# Transcriptome and proteome analysis reveal new insight into proximal and distal responses of wheat to foliar infection by *Xanthomonas translucens*

**DOI:** 10.1038/s41598-017-10568-8

**Published:** 2017-08-31

**Authors:** D. Garcia-Seco, M. Chiapello, M. Bracale, C. Pesce, P. Bagnaresi, E. Dubois, L. Moulin, C. Vannini, R. Koebnik

**Affiliations:** 1 IRD, Cirad, Univ. Montpellier, Interactions Plantes Microorganismes Environnement (IPME), 34394 Montpellier, France; 2 Dipartimento di Biotecnologie e Scienze della Vita, Università degli Studi dell’Insubria, via J.H. Dunant 3, 21100 Varese, Italy; 3 Université catholique de Louvain, Earth and Life Institute, Applied Microbiology Phytopathology, Louvain-la-Neuve, Belgium; 4 Council for agricultural research and economics (CREA) - Genomics Research Centre, via San Protaso 302, 29017 Fiorenzuola d’Arda, Piacenza Italy; 5 CNRS, Montpellier GenomiX, c/o Institut de Génomique Fonctionnelle, 141 rue de la Cardonille, Montpellier Cedex 34, France

## Abstract

The molecular details of local plant response against *Xanthomonas translucens* infection is largely unknown. Moreover, there is no knowledge about effects of the pathogen on the root’s transcriptome and proteome. Therefore, we investigated the global gene and protein expression changes both in leaves and roots of wheat (*Triticum aestivum*) 24 h post leaf infection of *X. translucens*. This simultaneous analysis allowed us to obtain insight into possible metabolic rearrangements in above- and belowground tissues and to identify common responses as well as specific alterations. At the site of infection, we observed the implication of various components of the recognition, signaling, and amplification mechanisms in plant response to the pathogen. Moreover, data indicate a massive down-regulation of photosynthesis and confirm the chloroplast as crucial signaling hub during pathogen attack. Notably, roots responded as well to foliar attack and their response significantly differed from that locally triggered in infected leaves. Data indicate that roots as a site of energy production and synthesis of various secondary metabolites may actively influence the composition and colonisation level of root-associated microbes. Finally, our results emphasize the accumulation of jasmonic acid, pipecolic acid and/or the downstream mediator of hydrogen peroxide as long distal signals from infected leaves to roots.

## Introduction

Plant infection by microbial pathogens is known to result in extended reprogramming at the transcriptomic, proteomic, metabolomic and epigenetic level; however, the mechanisms involved have for the most part not yet been deciphered. During infection, bacteria attempt to create a favorable environment for colonization and propagation whereas plants attempt to resist infection via the induction of an enormous amount of defense mechanisms. In the field of plant pathology, the interaction between (hemi)biotrophic pathogens and plants has been framed by the zig-zag model^1^. According to this model, the plant recognizes pathogen (or microbe)-associated molecular patterns, PAMPs (or MAMPs), which trigger a general plant defense response named PAMP-triggered immunity (PTI). Infection will result if the pathogen can efficiently suppress PTI via the delivery of “effector” proteins into the plant. However, the infection process can be reduced or halted if the plant recognizes these effectors via resistance (*R*) genes and triggers “effector-triggered immunity” (ETI); in this case effector proteins are referred to as avirulence (Avr) proteins^2^.

Long-distance signaling between the root and shoot and vice versa is an elemental part of plant responses to biotic and abiotic perturbations that keeps the whole plant alert against infection and mobilizes all resources available to the part of the plant under stress. In particular, the effects of aboveground pathogen attacks on belowground plant organs have gained considerable attention. A dramatic effect of shoot insect infestation on the root was found at the transcriptomic level^3, 4^. Moreover, in potato, the roots participate in the production of defense-associated compounds during foliar resistance to *Phytophthora infestans* ^5^.

Much less is known about aboveground–belowground interactions in the case of plant–microbe interactions. In this field, studies have been primarily orientated towards understanding hormonal responses. Indeed, plant hormones, as Jasmonic acid (JA), salicylic acid (SA), ethylene (ET), and abscisic acid (ABA) play an essential task in plant-microbe interactions. responses^6, 7^.

However, the molecular mechanisms of these pathways still remains unknown^8^. Moreover, there are important differences in hormonal networks between different plant species, such as *Arabidopsis* versus rice^8, 9^, which makes it difficult to decipher a general global plant response at the hormonal level.

Among phytopathogenic bacteria, members of the genus *Xanthomonas* are found all over the world and can infect a plethora of economically important dicot and monocot crop plants, causing a broad variety of diseases. For instance, *Xanthomonas citri* pv. *citri* and *Xanthomonas campestris* pv. *campestris* are major pathogens of citrus and cultivated brassica and radishes, respectively^10^. For monocots, *Xanthomonas oryzae* pv. *oryzae* and *Xanthomonas oryzae* pv. *oryzicola* leads to bacterial blight and leaf streak, respectively, which are among the most serious diseases of rice and constrain its production in Asia and Africa^11, 12^. *Xanthomonas translucens* is another important monocot pathogen that affects both forage grasses and small-grain cereals, such as wheat (*Triticum aestivum* L.)^13^. *Xanthomonas* has become a perfect model for studying bacterial infections and plant defense responses. The availability of genome sequences for dozens of plant-pathogenic *xanthomonas* ^14–16^ and their infected host plants^17–19^ has opened up the path for high-throughput “omics” analyses.

Indeed, several transcriptome studies have provided valuable data that have helped elucidate the complex genetic and hormonal networks induced by *Xanthomonas* infection in its host plants, including hormonal responses to *X. translucens* infection. For instance, Kölliker and coworkers compared transcriptomic changes in ryegrass upon pathogen challenge^20^. Genome-wide profiling of local and systemic transcript accumulation in barley shows gene expression changes in response to *X. translucens* pv. *cerealis* inoculation, and a clear difference between local and systemic responses. This approach has also been fruitful in showing the contribution made by WRKY and ERF-like transcription factors to plant responses^21^. Less work has been dedicated to studying the changes in the proteomes of plants upon inoculation with species of *Xanthomonas*; a two-dimensional electrophoresis approach has been used to explore the response of rice leaves to inoculation with different strains of *X. oryzae* ^22, 23^.

As far as we know, no studies have examined the changes that occur in the transcriptomes or proteomes of wheat inoculated with *Xanthomonas* species. Bread wheat (*Triticum aestivum* L.,) is one of the most important crops worldwide but it is generally considered that its hexaploid genome makes it difficult to study,. For these reasons, transcriptomic and proteomic analyses in wheat are particularly challenging and only a few studies have been performed.

The aim of this study was to obtain further insight into local and distant cellular events 24 h after the infection of wheat leaves by *X. translucens*. We integrated whole-transcriptome shotgun sequencing (RNA-seq) and shotgun nanoflow scale liquid chromatography-tandem mass spectrometry (LC-MS/MS) proteomics to get a global view of wheat responses during the infection process. The simultaneous analysis of leaves and roots allowed us to capture significant metabolic rearrangements triggered by leaf infection with *Xanthomonas* both in above- and belowground tissues and to identify common responses as well as specific alterations.

## Material and Methods

### Bacterial strains and plant inoculation

The *X. translucens* type strain CFBP 2054 (syn. ATCC 19319, DSM 18974, ICMP 5752, LMG 876, NCPPB 973; GenBank acc. no. LT604072.1) was used in this study. Bacteria were cultivated at 28 °C in Peptone sucrose agar (PSA) medium.

**Figure 1:**
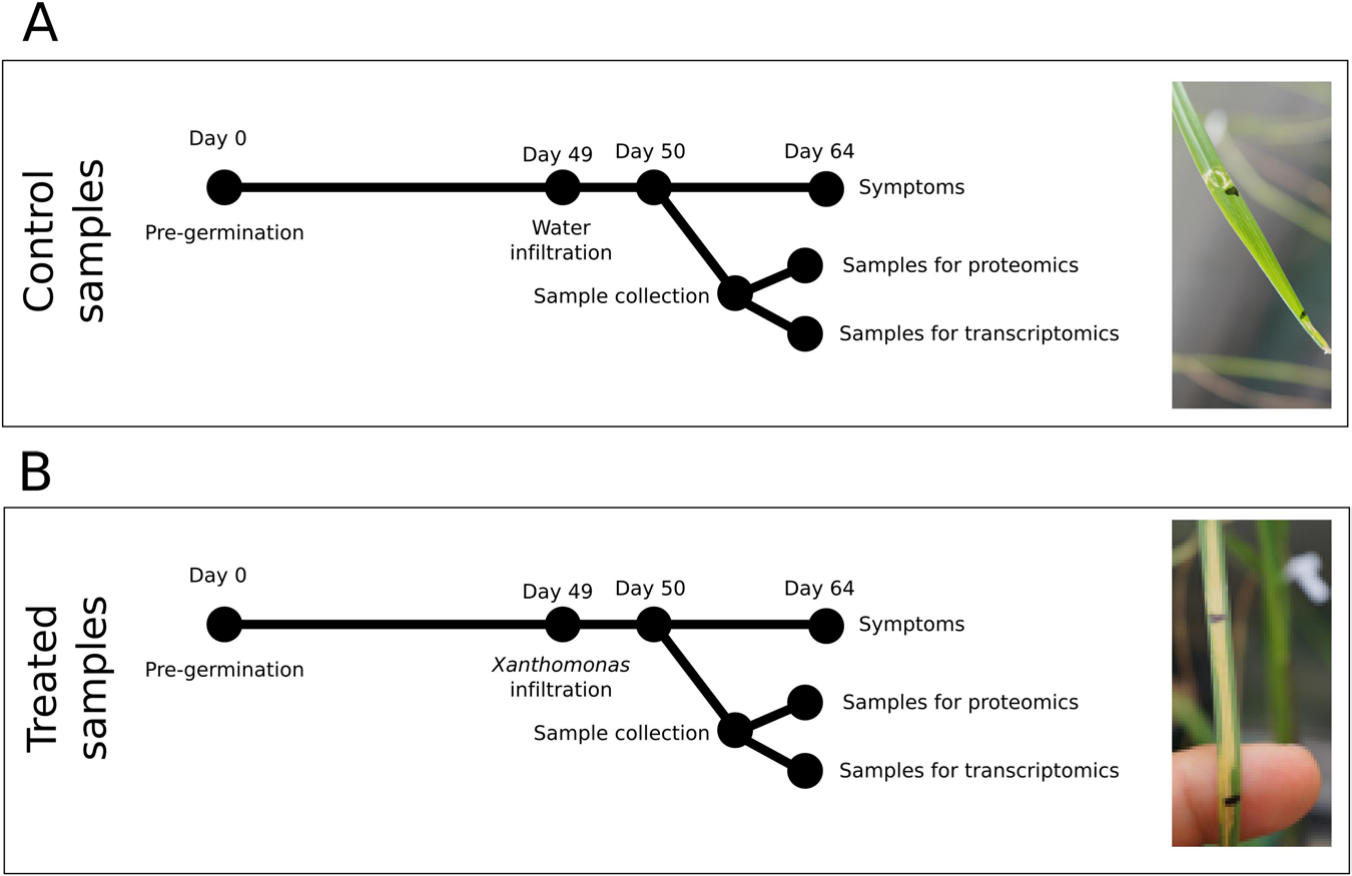
Schematic representation of workflow for (**A**) control samples and (**B**) –omics analyzed samples. 24 h post inoculation samples from leaves and roots were collected. In parallel, 14 dpi symptoms were assessed. The pictures show symptoms of inoculated leaves.

*Triticum aestivum* cv. Chinese Spring plants were maintained in a growth chamber with cycles of 12 h of light at 21 °C and 50% relative humidity (RH) and 12 h of dark at 21 °C and 50% RH. Leaves of 49 days-old plants were infiltrated with a bacterial suspension in water with an optical density at 600 nm (OD_600_) of 0.5 using a needleless syringe. Plants inoculated with water were used as controls. For transcriptomic and proteomic analyses, leaves and root tissues were harvested 1 day post-inoculation (dpi), when symptoms were not visible yet. In parallel, several infected plants were kept in the growth chamber to follow symptom formation over the next two weeks (Fig. 1). The infiltrated zone was collected as previously described^24^. Three biological replicates per treatment were performed, and each with pooled leaves from two independent plants per replicate. Once pulverized in liquid nitrogen, each sample was split into two aliquots, for protein and RNA extraction respectively.

### RNA-seq transcriptome sequencing

RNA-seq transcriptome sequencing was performed on leaves and roots. Samples were ground to a fine powder in liquid nitrogen and total RNA was isolated (TRI Reagent^®^ (MRC, Cincinnati, OH))^25^. Then, it was treated with DNase and RNA quality was confirmed with Experion™ Automated Electrophoresis System, Nanodrop™ and gel electrophoresis. Total RNA was used for mRNA preparation, fragmentation and cDNA synthesis.

The preparation of mRNA libraries was done using the TrueSeq Stranded mRNA Sample Preparation kit (Illumina Inc., San Diego, CA). The library quality validation (concentration and length of DNA fragments) was performed by Fragment Analyzer using the standard and high sensitivity DNA kits (Agilent Technologies, Santa Clara, CA) and by qPCR (LightCycler 480; Roche Diagnostics, Meylan, France). MGX-Montpellier GenomiX platform made RNA-seq library preparation and sequencing using a HiSeq. 2500 instrument (Illumina Inc.). Six libraries were sequenced per lane (around 30 millions reads per replicate), with 100 nucleotides sequence length per read.

### Bioinformatic analysis of transcriptome data

Mapping and alignment of sequence reads was done with Burrows-Wheeler transform using the BWA-MEM algorithm, which automatically chooses between local and end-to-end alignments, supports single reads and performs chimeric alignment^26^. The BWA-MEM algorithm is robust to sequencing errors and applicable to a wide range of sequence lengths from 70 bp to a few megabases. Specifically, reads were aligned to *Triticum aestivum* coding sequences (CDS; IWGSC1.0 + popseq. 29) and non-coding RNA (ncRNA). Subsequently, sequence counts were collected by Samtools idxstats filtering of bam alignment files for reads with MAPQ ≥ 3.

Differentially expressed genes (DEGs) were called via DESeq2 1.10.0 Bioconductor package using local fit and betaPrior parameter set to False. DESeq2 implements differential expression analysis based on the Negative Binomial distribution. Independent filtering was enabled^27^. A false discovery rate (FDR) threshold of 0.05 was set for DEG calling^28^. Sample clustering and principal component analyses were performed upon variance stabilizing transformation of expression data (DESeq2 package). Transcripts were called as differentially expressed when the FDR-BH adjusted *p* values were below 0.05 and fold-changes over 2.

The transcriptomics data have been deposited in the European Bioinformatics Institute (EMBL-EBI) with the dataset identifier E-MTAB-5891.

### Protein extraction and digestion

The used protocol for total protein extraction was based on a method combining trichloroacetic acid (TCA)/acetone precipitation with SDS and phenol extraction, which works well with both root and leaf samples (Wu *et al*. 2014). Proteins were digested and prepared for mass spectrometry using the FASP method^29^. Protein digestions were conducted overnight at 37 °C with 1:100 trypsin to sample (Promega, Dübendorf, Switzerland). After digestion, the liberated peptides were collected by centrifugation and a second trypsin digestion was performed for 4 hours at 37 C with 1:20 trypsin to sample to enhance the amount of peptide recovery. The liberated peptides were collected as previously described.

### Liquid chromatography-mass spectrometry analysis

Mass spectrometry analysis was performed on a QExactive mass spectrometer coupled to a nano EasyLC 1000 (Thermo Fisher Scientific Inc., Waltham, MA). Solvent composition was 0.1% formic acid and 0.1% formic acid, 99.9% acetonitrile, respectively for channel A and B. For each sample, 4 μL of peptides were injected on a self-made column (75 μm × 150 mm) packed with reverse-phase C18 material (ReproSil-Pur 120 C18-AQ, 1.9 μm; Dr. Maisch GmbH, Ammerbuch, Germany). The flow rate was 300 nL/min by a gradient from 2 to 35% B in 80 min, 47% B in 4 min and 98% B in 4 min. The order of the sample acquisition was randomized. Mass spectrometer operated in data-dependent mode (DDA), full-scan MS spectra were acquired between 300–1700 m/z and the twelve most intense signals per cycle were fragmented. HCD spectra were acquired at a resolution of 35,000 using a normalized collision energy of 25 and a maximum injection time of 120 ms. 50,000 ions was the set fot the automatic gain control (AGC). Charge state screening was enabled and singly and unassigned charge states were rejected. Precursor masses selected from previous measurement were excluded for 30 s, and 10 ppm was used as exclusion window. The samples were acquired using internal lock mass calibration on m/z 371.1010 and 445.1200.

### LC-MS/MS data analysis

Mass spectrometer raw files were analyzed using MaxQuant (version 1.5.3.28) with the match between runs, matching time window of 2 min and label free quantification (LFQ) options selected. LFQ intensities are the output of the MaxLFQ algorithm (Cox *et al*. 2014). They are based on the (raw) intensities and normalized on multiple levels to ensure that profiles of LFQ intensities across samples accurately reflect the relative amounts of the proteins.

Tandem MS spectra were searched against UniProt *T. aestivum* (Version 2015-10, 100,800 entries) and Uniprot *X. translucens* (Version 2015-10, 29,847 entries). Trypsin/P was chosen as the protease, cysteine carbamidomethylation was set as fixed modification, and oxidation of methionine and acetylation of the N-terminal as variable modifications. Peptide tolerance was set to 20 ppm, while MS/MS tolerance was set to 0.5 Da. False discovery rate (FDR) was set at 1% both for peptide-spectrum matches (PSMs) and proteins. Only PSMs with a minimum length of 7 amino acids were kept.

### Proteomics data processing

Once identified, all protein groups without contaminants and decoy entries as well as groups without “only identified by site” were chosen. Resulting protein groups were considered as identified in the following cases: (i) All replicates in each condition contain a valid quantitative value; (ii) control condition contains 2 or 3 valid values and treated condition can contain any value, and (iii) treated condition contains 2 or 3 valid values and control condition contain any value. This process led to many cases of missing values for one or the other condition. Indeed, proteomics data analysis is often hampered by missing values. There are two types of missing values: the ones resulting from absence of detection – missing at random (MAR) – and the ones resulting from their absence – missing not at random (MNAR). The latter are biologically relevant. Missing data imputation has been conducted using the *R* package ‘imputeLCMD’ (https://cran.r-project.org/web/packages/imputeLCMD/). For the MAR values, we used the KNN imputation (Troyanskaya *et al*. 2001), while for the MNAR values, we used the stochastic minimal value approach. Proteins were considered differentially expressed when the adjusted *p* values were below 0.05 and the logarithmic fold change was over 0.5 or when the logarithmic fold change was over 2. For *p* value correction we used the Bioconductor package called qvalue^30^. To obtain functional annotation, sequences of identified proteins were mapped using The Arabidopsis Information Resource, version 10 (http://www.arabidopsis.org), by BLASTX with *E* value cut-off ≤e^−10^).

The mass spectrometry proteomics data have been deposited to the ProteomeXchange Consortium via the PRIDE partner repository with the dataset identifier PXD006870.

### MapMan analyses

To display the large datasets onto diagrams of metabolic pathway, we used the MapMan tool^31^. Figures were generated by exporting DESq2-normalized expression data to the MapMan application (http://mapman.gabipd.org). CDS were assigned to MapMan bins via IWGSP_MIPSv2.2.txt mapping file, as available in the MapMan repository (http://mapman.gabipd.org/web/guest/mapmanstore).

For the proteomic analysis, we compared sequences of the all differentially expressed proteins against the TAGI database (DFCI TGI – http://occams.dfci.harvard.edu/pub/bio/tgi/data/) using the NCBI Blast+application suite (http://ftp.ncbi.nlm.nih.gov/blast/executables/blast+/LATEST/), to obtain the best matching *T. aestivum* gene identifier for each protein in our lists. All aligned genes had an identity score >80%. Every protein identifier was thus converted to a corresponding gene ID in the TAGI r12 database. MapMan v3.5.1 was loaded with *T. aestivum* TAGI r12 database downloaded from the MapManStore (http://mapman.gabipd.org/web/guest/mapmanstore) website. Our listing IDs were loaded into MapMan after the above-mentioned conversion along with fold change (FC) values for each entry. All MapMan diagrams were analyzed using a color scheme and a scale level that allowed an easy visualization of up- (blue) or down- (red) regulated genes or proteins.

### Detection of carbonylated proteins

The proteins were extracted as above described^32^ and 20 μg of proteins were derivatized with 10 mM 2,4-dinitrophenylhydrazine (DNPH) in 2 M HCl. 2 M HCl was added to the negative control. The mixture was incubated for 30 min at and then neutralized by adding 1 volume 2 M Tris, 30% glycerol. After a 12% SDS-PAGE, proteins were transferred to polyvinylpyrrolidone (PVP) membrane (Serva Electrophoresis GmbH, Heidelberg, Germany) and the oxidatively modified proteins were detected using anti-DNPH antibodies (antidinitrophenyl-group antibodies; Sigma, USA) and visualized by SuperSignal detection kit (, Pierce Biotechnology, Rockford, IL, USA). Duplicate gel was stained by Colloidal Coomassie Brilliant Blue (CCBB). Alternatively, gels were stained with Bio-Safe Coomassie (Bio-Rad) and then processed for immunoblotting. GS-800 (Bio-Rad) and ImageJ software (http://imagej.nih.gov/ij/) were used to acquire and analyse gels and immunoblot images.

## Results

### Transcriptome analysis in response to *X. translucens* infection

To gather information about the transcriptional events involved in the wheat response to *X. translucens* infection, RNA-seq was performed on roots and leaves of 7-week old plants of the cultivar Chinese Spring 24 hours after treatment with the pathogen. RNA from each control and infection condition was sequenced in triplicates, with 39 million reads on average per replicate, and a minimum of 26 million and maximum of 52 million (Supplementary Table S1). The median and the first quartile of quality scores were above 30, indicating a quality higher of 99.9% in all samples.

**Figure 2:**
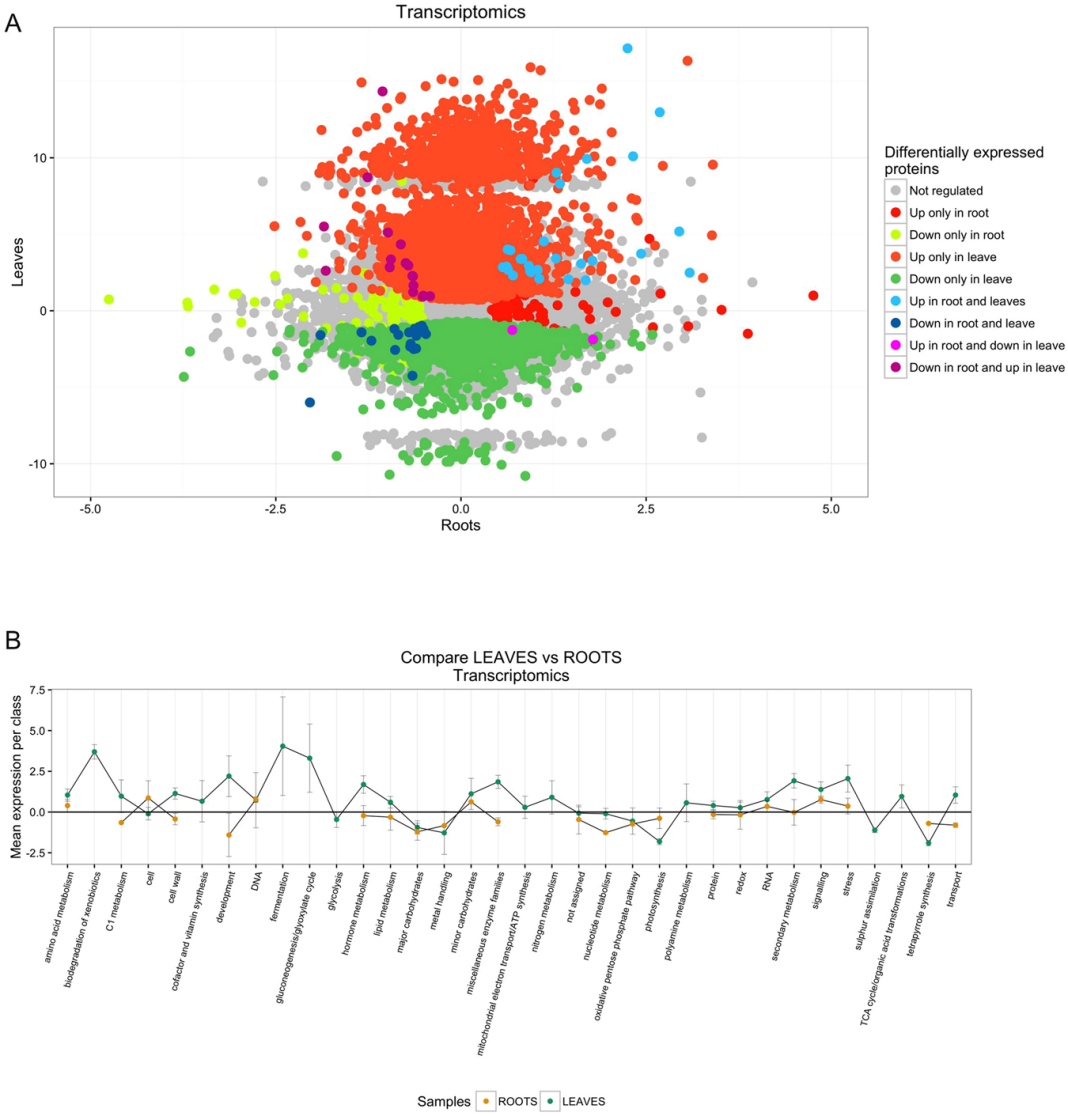
Transcriptomics data. (**A**) Overlap between DEGs presents in roots and leaves. (**B**) Overlap between MapMan categories identified by transcriptomics comparisons between leaves and roots. In order to draw the plot, the gene expression in all MapMan categories have been averaged.

Analysis of the mapped reads (Supplementary Table S2) showed that the infection of *X. translucens* led to significant differential expression of 9,320 wheat transcripts in leaves, of which 3,765 were down- and 5,555 were upregulated. Moreover, we identified 324 wheat transcripts in roots, including 200 downregulated genes and 124 upregulated genes (Supplementary Table 3). In order to identify common trends of regulation, we compared the Differentially Expressed Genes (DEGs) between leaves and roots. Most of the DEGs were tissue specific (Fig. 2A), but a small percentage of genes was regulated both in leaves and roots (7449 DEGs), mostly in the same direction (Supplementary Table S4).

9211 DEGs in leaves and 315 in roots were assigned to different metabolic pathways using Mapman. We were able to classify 8968 data points in leaves and 332 data points in roots (Fig. 2B and Supplementary Table S5). Our data revealed how complex and still unknown the plant-pathogen interactions are, as exemplified by the 2208 data points that were detected without a known function (unknown or no ontology).

### Proteomics changes in early response to pathogen infection

For a more complete picture of the molecular changes of wheat roots and leaves after inoculation with *X. translucens* we combined quantitative proteome profiling with RNA-seq analysis. In total, 2721 protein groups were consistently identified and quantified in the leaves (Supplementary Table S6). 366 (13%) proteins resulted regulated by *X. translucens* infection in leaves, equally divided between up- and downregulation. In order to assess the analytical reproducibility of our analysis, correlation values between control and treated samples have been calculated. The range of correlation between control samples was 0.975 to 0.989, the range between treated samples was 0.976 to 0.987, while the correlation range between the two studied conditions was between 0.870 and 0.906. All samples were very correlated, but the two conditions revealed some differences.

**Figure 3:**
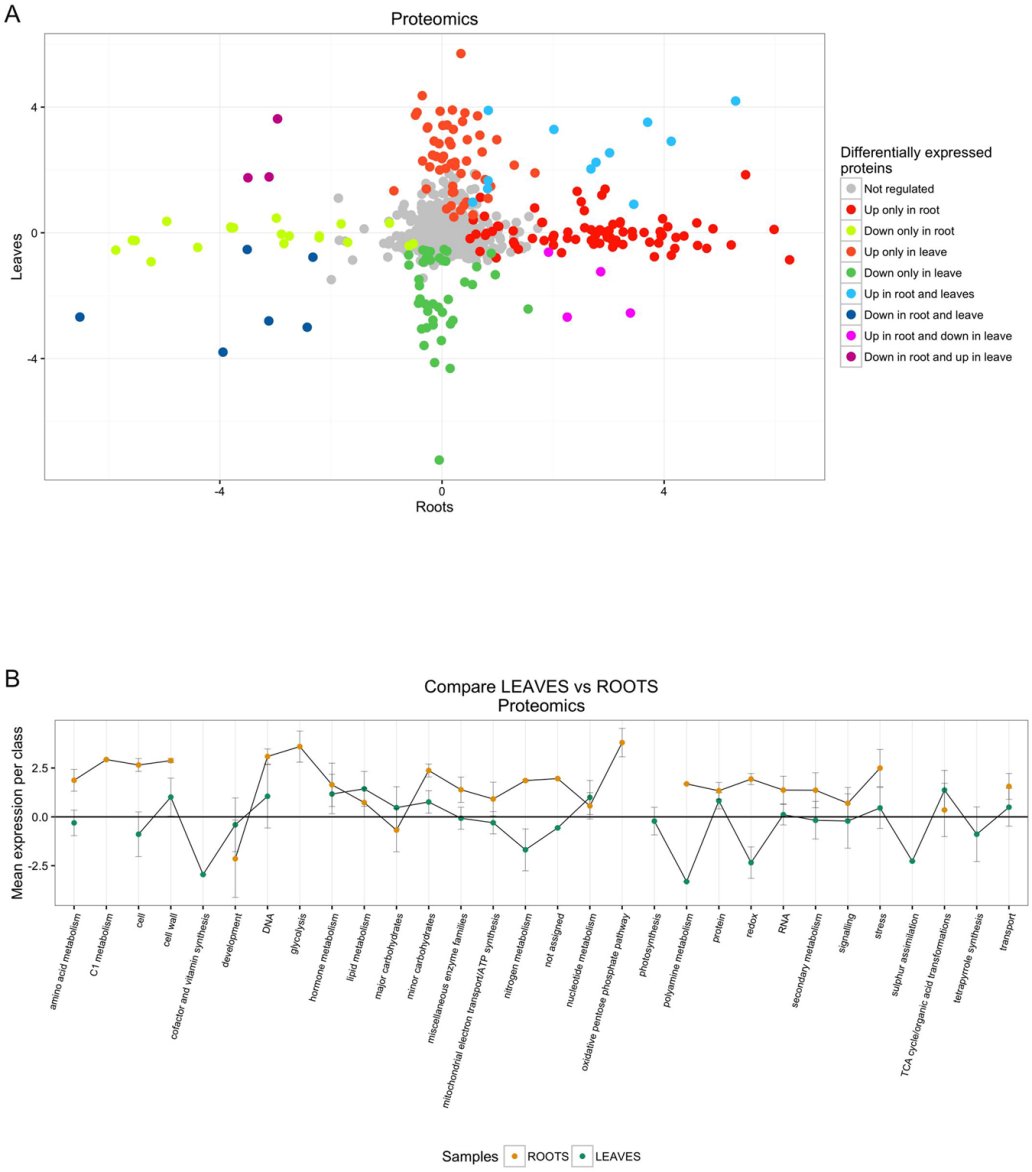
Proteomics data. (**A**) Overlap between DEPs presents in roots and leaves. (**B**) Overlap between MapMan categories identified by proteomics comparison between leaves and roots. In order to draw the plot, protein expression levels in all MapMan categories have been averaged.

In roots, a total of 2446 protein groups were consistently identified and quantified (Supplementary Table S6). 357 (15%) different proteins were affected in roots by *X. translucens* infection of leaves. Among them, 274 (12%) were upregulated, while 83 (3%) are downregulated. All the identification data are listed in Supporting Information Table S6. Despite the short treatment of leaves with *X. translucens*, the root samples clustered in two distinct groups: control samples and treated samples (data not shown). The range of correlation values between control samples was 0.982 to 0.990, the range between treated samples was 0.977 to 0.991, indicating that the mass spectrometric analysis had robust reproducibility. The correlation values between the two studied conditions were 0.873 to 0.888. The Pearson coefficients showed that all samples were very highly correlated, but the two conditions revealed some differences as well. Leaf and root identifications were compared in order to understand how many proteins were present in both datasets. The common proteins were 1259, while 1462 have been only identified in leaves and 1187 exclusively in roots (Fig. 3A). Despite the consistent overlay in the protein identification only 12 were upregulated and only 6 were downregulated both in root and leaf samples (Supplementary Table S7).

### Functional analysis of Differentially Expressed Proteins (DEPs)

Proteins with significant changes in abundance (357 in leaves and 366 in roots respectively) were assigned to at least one MapMan^31^ functional category and the proteins with unknown function were assigned to MapMan BIN 35. Both in leaves and roots the DEPs were mapped in the same categories (except for photosynthesis for leaves and DNA synthesis for roots). The most represented pathways were protein metabolism, RNA transcription and processing, enzyme families, redox, biotic stress, secondary metabolism and amino-acid metabolism. However, root samples showed a significant upregulation in all categories while in the leaves all categories were approximately equally divided between up- and downregulation. Moreover, Mapman visualization reported that a large proportion (25% and 29%) of DEPs was related with biotic stress in leaves and roots respectively (Fig. 3B and Supplementary Table S8). Among them, about 56% were upregulated in leaves and 77% in roots.

## Discussion

**Figure 4:**
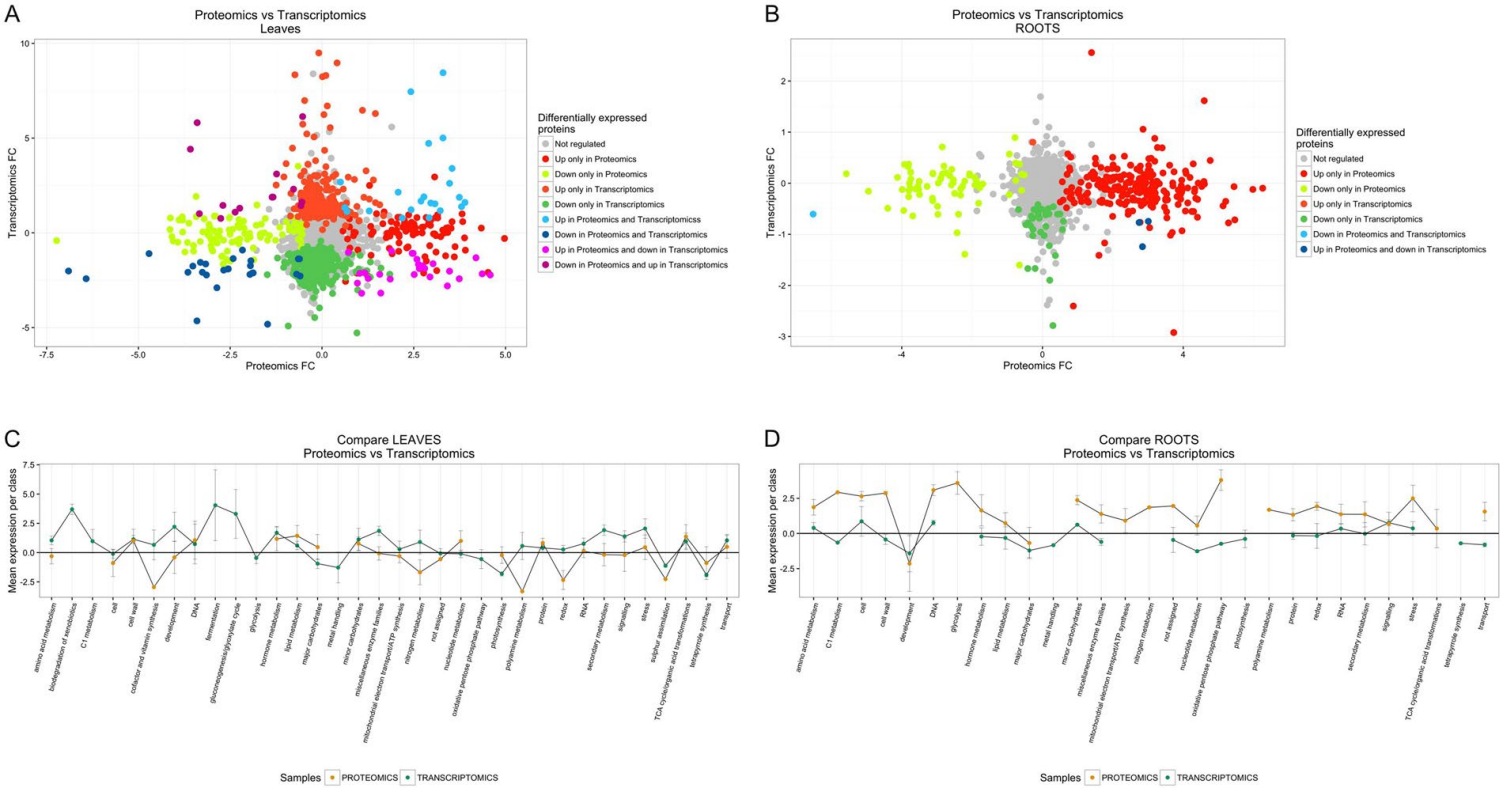
Overlap of proteins identified by both proteomics and transcriptomics. (**A**) Leaves, (**B**) Roots. MapMan overlap between proteomics and transcriptomics samples. (**C**) Leaves, (**D**) Roots.

To date, no study has simultaneously addressed the molecular events triggered by *Xanthomonas* infection in proximal and systemic distal plant tissues. Here, we investigated the global transcriptomic and proteomic changes induced by *X. translucens* 24 hpi in leaves and roots of wheat (Fig. 4). In this section, we discuss the most interesting pathways affected by *X. translucens* in both infected leaves and roots.

### *X. translucens* infection activates a complex defense response at the site of infection

The first phase of the PTI response relies on the recognition of pathogen-derived molecules by pattern recognition receptors (PRRs). Transcriptomic data showed that a large number of receptor kinases were upregulated at the infected site (106 DEGs). Among these, we identified FLS2 and EFR, which are well-characterized leucine-rich repeat receptors that recognize bacterial flagellin and EF-Tu elongation factor, respectively. In addition, the upregulation of some receptor-like cytoplasmic kinases (RLCKs), such as Brassinosteroid signaling kinase 1 (BSK1) and PTI Compromised Receptor-like Cytoplasmic Kinase 1 (PCRK1), may contribute to PTI in response to *X. translucens*.

Two putative LysM-containing receptor-like kinase homologs of Arabidopsis LYK5 and CERK1, essential in the perception and signal transduction of the fungal chitin oligosaccharide elicitor, were upregulated by bacterial infection. In addition to its function in anti-fungal immunity, CERK1 is also involved in the perception of bacterial peptidoglycans^33^ and is required for resistance to bacterial infection^34^. These findings suggest the presence of an overlapping receptor system in wheat, as previously suggested in rice^35^. The recognition of different microbial ligands may contribute to a more rapid achievement of the signaling threshold needed for PTI activation.

There is increasing evidence that other receptor kinases, some of which were upregulated, act as additional plant immune receptors. Of these, several plant lectin and cysteine receptor kinases (CRKs) are upregulated upon perception of flagellin and act coordinately to enhance plant immune responses^36–38^. Expert manual data analysis also revealed upregulation of the Clavata 2 (CLV2) transcript. Very recent data indicate that CLV2 is implicated in plant immunity^39^. Six DEGs encoding leucine-rich repeat receptor kinases involved in the perception of sulfated peptide PSY1 were strongly overexpressed at the site of infection. The activation of these receptors leads to the downregulation of SA-related responses and upregulation of JA signaling after infection by a biotrophic pathogen. PSY1 also promotes plant growth^40^. Our data confirm that PSY1 signaling is important for the integration of plant growth with defense responses.

Finally, several wall-associated receptor kinases (WAKs) were upregulated. One of these, WAK1, recognizes oligogalacturonides (OGs), which are degradation products of the plant cell wall released upon pathogen infection, and triggers immune responses^41^.

Our experiments suggest that the blue-light photoreceptors PHOT1 and CRY1 were downregulated in response to infection. Both genes are involved in blue light-induced stomatal opening and their downregulation may lead to stomatal closure to restrict bacterial invasion. Upregulation of three members of the Plant U-box family of ubiquitin E3 ligases (PUB22, PUB23 and PUB12), which are involved in proteasomal degradation of PRR complexes^42^, reveals an additional layer of complexity. Protein turnover may contribute to tight control of PRR abundance to avoid inappropriate activation.

Overall, these data demonstrate the amplitude and complexity of the PRR response to a single microbial species. The accumulation of plasma membrane receptors may lead to stronger and faster immune responses against subsequent pathogen attack (data not shown).

### Diverse signaling pathways are stimulated at the infection site

Signaling events observed after pathogen recognition are mediated by ion fluxes across the plasma membrane, such as an influx of Ca^2+^ into the cytosol, a burst of ROS, and the activation of protein kinase cascades. Genes encoding calmodulin-binding proteins, calcineurin proteins, calcium ATPase, and glutamate receptor-like channels (GLURs) were upregulated at the site of infection. These proteins constitute a network that can induce and maintain intracellular Ca^2+^ levels to trigger signaling pathways. For example, GLURs were proposed to act as amino acid (AA)-gated calcium channels to perceive changes in the apoplastic AA concentrations resulting either from cell damage or from PAMP-induced exocytosis^43^. Our data confirm that GLURs can contribute to PTI activation in parallel with PRRs (data not shown).

Upregulation of some calcium-dependent protein kinases (CDPKs) was also observed. CDPKs were proposed to function in multiple plant signal transduction pathways downstream of Ca^2+^ cytoplasmic elevation. CPK1 can phosphorylate phenylalanine ammonia lyase (PAL), a key enzyme in pathogen defense, while CPK3 functions in guard cell ion channel regulation.

MAPK cascades are known to be implicated in plant immunity. In this study, we observed the upregulation of EDR1, MAPKKK5, MAPKKK15, MAPKKK3, MAPKK2, MAPKK5, MPK9, MPK3, and MPK4. EDR1 is a MAPKKK serine/threonine-protein kinase that is involved in the regulation of a MAP kinase cascade (probably including MPK3 and MPK6) that negatively regulates SA-dependent defense responses, ABA signaling, and ET-induced senescence^44^.

PTI can be attenuated by effector molecules secreted into plant cells by microbial pathogens^45^. Plants have evolved a class of immune receptors, encoded by disease resistance (*R*)-genes that recognize the presence or activity of effectors and induce the effector-triggered immunity (ETI) response. In this study, 149 DEGs that encoded disease resistance genes, e.g. *RPM1* and *RPS2*, were differentially regulated at the site of infection. Although ETI constitutes a stronger immune response that is often associated with the hypersensitive reaction (HR), a form of programmed death of plant cells (PCD) at infection sites, the ETI and PTI gene expression signatures were largely similar. Metacaspase AMC1, a positive regulator of PCD^46^, and four proteins involved in suppression of cell death (Bax inhibitor 1, ATDAD1, CAD1, and BONZAI 3) were upregulated concurrently. These data indicate that plant cell death is simultaneously stimulated and repressed, maintaining cellular homeostasis. This may explain the lack of symptoms at the pathogen entry site 24 hpi.

### Chloroplasts play a central role against *X. translucens* attack

Some studies suggest that photosynthesis is essential for the defense response^47^. Our transcriptomic and proteomic data showed that *X. translucens* infection had a substantial effect on components of the photosynthetic apparatus and on chloroplast biogenesis in the infected leaf. Indeed, a very large number of genes and proteins related to photosystem II, chlorophyll and carotenoid biosynthesis were significantly downregulated in treated leaves compared with control leaves. These included transcripts encoding the FLU and GUN4 Arabidopsis homologs required for tetrapyrrole biosynthesis regulation. Moreover, the Calcium-sensing protein (CAS) was deregulated. These latter proteins are involved in retrograde signaling and their impairment leads to ROS accumulation in the chloroplast^48^. Finally, the transcription factor NF-YB, which regulates the expression of nuclear-encoded chloroplast-targeted genes and the normal development of chloroplasts^49^, was affected in treated leaves at both the RNA and protein levels.

Transcriptomic data showed that some Calvin cycle genes were downregulated, while proteomic analysis showed accumulation of four key enzymes of the cycle in infected leaves. A similar lack of correlation between levels of mRNA and protein involved in photosynthesis was reported by Gohre *et al*.^50^. These data suggest that the plant attempts to maintain carbon assimilation stable during pathogenic attack.

In infected leaves, the expression levels of some genes and proteins that have important roles in chloroplast sulfate assimilation, including sirohydrochlorin ferrochelatase B (SirB), ATP-sulfurylase, and cysteine synthases, were significantly decreased. Cysteine acts as a precursor or donor of reduced S for a range of S-compounds, such as methionine (Met), and glutathione (GSH). Several enzymes involved in Met salvage, a Met recycling pathway, were upregulated. Met regeneration may play an important role in sustaining the production of ET in infected tissues (see below).

Siroheme is a cofactor of ferredoxin-nitrite reductase (NiR), and, accordingly, the level of NiR was significantly downregulated in infected leaves. At the transcriptomic level, two nitrate reductases were downregulated and some DEGs corresponding to ammonium transporter (AMT2) genes were strongly induced in leaves. By contrast, in the roots, proteomic data showed that NiR, AMT2, and one high-affinity nitrate transporter were upregulated.

Transcription of chloroplastic precursors of the glutamine synthase GS2 and the ferredoxin-dependent glutamate aminotransferase (Fd-GOGAT), involved in primary nitrogen assimilation, also decreased, while the genes encoding cytoplasmic GS and NADH-GOGAT were strongly induced. Finally, the observed increase in glutamate dehydrogenase transcripts indicated that glutamate was used as a source of energy and carbon in infected leaves. Overall, these data indicate that 1) the chloroplast is a strategic battlefield during pathogen attack and, confirming recent research, is also a key component of the plant immune response^51^; 2) remobilization is the main source of nitrogen in the infected leaf, whereas the root is involved in primary N assimilation.

### ROS and redox regulation are important contributors to the wheat response to *X. translucens*

The production of ROS is one of the first responses that occurs after pathogen recognition^52, 53^. Accordingly, our datasets showed the upregulation of NADPH/respiratory burst oxidase protein D (RbohD) and of extracellular peroxidases, major regulators of the apoplastic oxidative burst around the infection site that are also involved in cross-linking cell wall components^54, 55^.

In infected leaves, 35 of 45 DEGs encoding ascorbate peroxidases (APXs) were strongly upregulated. APX enzymes play a key role in catalyzing the conversion of H_2_O_2_ to H_2_O, using ascorbate or GSH as a specific electron donor. Conversely, at the protein level, there was abundance of only one APX, while levels of three APX proteins decreased. Two of the three downregulated proteins were stromal and thylakoid membrane-bound APXs: sAPX and tAPX, respectively. These key enzymes are involved in H_2_O_2_ scavenging in chloroplasts. tAPX reduction enhances nitric oxide-induced cell death^56^ and activates defense responses^57^. Our findings support the involvement of tAPX in the response to pathogens, probably through slight changes in the H_2_O_2_ concentration in chloroplasts.

Several chloroplast thioredoxins were downregulated in leaves at both the RNA and protein levels. The major function of these proteins is to reduce the disulfide bonds of their substrate proteins and maintain cellular redox homeostasis. Glutathione S-transferases (GSTs) were mostly upregulated at proteomic and transcriptomic levels.

Proteomic results indicated that some enzymes involved in ROS detoxification and cell redox homeostasis, such as APXs, GSTs, and thioredoxins, also accumulated in the roots of infected plants. Some SODs were upregulated at the transcriptomic and proteomic levels. The response of peroxidases was less evident, because nine of the ten peroxidases identified were downregulated at the transcriptomic level, while proteomic analysis showed the accumulation of five peroxidases among the seven that were identified. We can speculate that the downstream mediators of foliar pathogen attack induce mild oxidative stress in roots, enhancing the accumulation of proteins involved in ROS scavenging.

**Figure 5:**
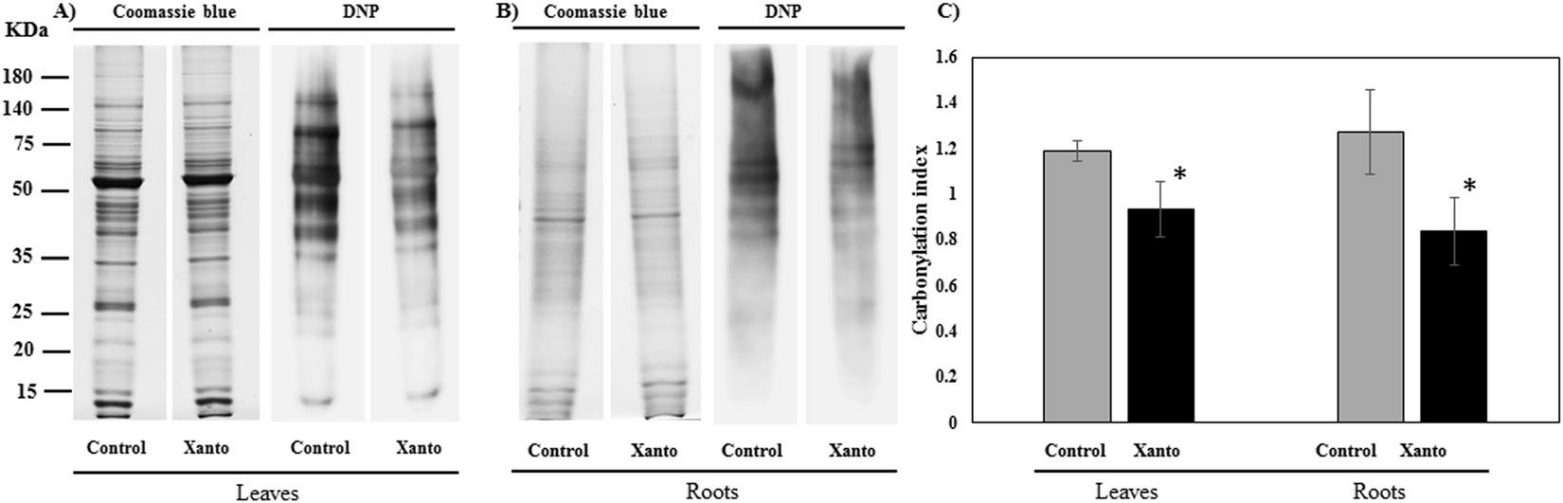
Protein carbonylation profiles of wheat plants after *Xanthomonas* infection: (**A**) leaves protein stain (left), anti-DNP (2,4-dinitrophenol) immunoassay (right); (**B**) roots protein stain (left), anti-DNP (2,4-dinitrophenol) immunoassay (right); (**C**) relative protein carbonylation values (referred to the control sample) expressed as the carbonylation index, after normalization for protein amounts. Data (means ± SD, n = 3) were subjected to one-way analysis of variance (ANOVA). The asterisks indicate significant differences at the 5% level using Tukey’s test.

We studied the accumulation of oxidative modified polypeptides by conducting an immunoblot analysis of carbonylated proteins. Protein carbonylation, which is irreversible, is one of the most harmful oxidative protein modifications and is considered a major hallmark of oxidative damage^58^. The extent of protein carbonylation was lower both in leaves and in the roots of plants exposed to *X. translucens* than in control samples (Fig. 5). This result indicates that the antioxidant defense system was able to cope with the oxidative stress induced by bacterial infection at 24 hpi, even in infected leaves.

### *X. translucens* infection induces metabolic changes in proximal and distal plant tissues

The decrease in photosynthesis leads to a metabolic shift of infected tissues from source to sink. The induction of cell wall invertases (cw-Inv) and hexose and sucrose transporters is considered to be the primary cause for the formation of a sink at the infection site^59^. Of 22 DEGs encoding transcripts for vacuolar and apoplastic invertases, 14 were upregulated, while at the proteomic level one cw-Inv protein was downregulated. Sonnewald *et al*. demonstrated that *X. campestris* pv. *vesicatoria* suppressed cw-Inv activity in a T3SS-dependent manner^60^. Invertases cleave sucrose into glucose and fructose, thereby preventing sucrose export from infected cells. Furthermore, 25/43 DEGs coding for sugar transporters were overexpressed. One of these was a transcript encoding a STP13 homolog. It was recently demonstrated that STP13 reduces sugar content in the Arabidopsis apoplast, resulting in limited bacterial proliferation^61^.

Hexokinase (HXK), the best investigated glucose sensor, can show the energy status of the cell and induce subsequent signaling cascades to modulate cellular metabolism in response to carbon status^62^. Increasing evidence indicates that hexoses help defense responses^59^. Some HXKs accumulated in infected leaves, possibly contributing to the generation of ROS and the activation of PR genes^63^. Several other sugar kinases, such as galactokinase1 (GAL1) and arabinose kinase 1 (ARA1), were overexpressed in infected leaves; however, it is not yet clear whether these enzymes have signaling functions similar to that proposed for HXK.

This study showed that AA anabolic and catabolic pathways were also modified in infected leaves. Regulation of AA content and transport is critical for plant adaptation to carbon and nitrogen status, development, and defense^64^. Several enzymes involved in aspartate, alanine, proline, asparagine, arginine, methionine, lysine, phenylalanine, tyrosine, and tryptophan biosynthesis were induced at the site of *X. translucens* infection, as shown by transcriptomic and proteomic analysis.

Glutamine, asparagine, arginine, and proline may be involved in N recycling, remobilization, and translocation in infected leaves. Accordingly, some DEGs coding for an asparagine synthetase, ASN1, whose expression responds to the level of sugar in the cell^65^, were overexpressed. Conversely, DEGs coding for asparaginase were downregulated. Many molecules involved in plant defense are derived from AA, such as ET from methionine and phytoalexin, anthocyanin, and SA from phenylalanine. Some DEGs encoding AA metabolism enzymes that are involved in plant immunity were overexpressed. Of these, ornithine-d-aminotransferase and aspartate oxidase were previously shown to contribute to pathogen-induced ROS production and HR^66^.

Finally, some DEGs corresponding to AGD2-like defense response protein 1 (ALD1) homologs (involved in lysine degradation) were strongly overexpressed. *ALD1* transcript levels rise both locally and systemically in pathogen-inoculated Arabidopsis^67^. The lysine catabolite pipecolic acid (Pip) plays an important role in defense amplification and priming that allows plants to acquire immunity at the systemic level^68^. Recently, it was proposed that FMO1, whose transcripts were upregulated in our experiments, acts as a critical mediator for Pip-activated responses^69^. We speculate that Pip could function as a mobile element in shoot-root communication during foliar attack by *X. translucens*.

Foliar infection induced a metabolic change in the roots. Briefly, a SnRK1 kinase that was involved in the regulation of nitrogen and in the metabolism of sugar, and some genes and proteins related to AA metabolism, were upregulated at the RNA and protein levels. Some mitochondrial membrane metabolite transporters and some components of the respiratory chain complex were also upregulated. Components of the mitochondrial permeability transition alter their abundance during the plant basal defense response^70^. These data suggest that mitochondria in roots respond to aboveground attack and may supply energy for metabolic changes in both roots and leaves.

Overall, these data indicate the necessity of maintaining energy and solute homeostasis for cell protection during pathogen progression both in proximal and distal plant tissues.

### Secondary metabolism and defense-related proteins

Consistent with previous research, our datasets showed that key enzymes of the phenylpropanoid pathway were triggered during pathogen attack^71, 72^. The cell wall is one of the most important barriers against pathogens, and lignification is important in plant defense^73^. This lignification was very clear at the transcriptomic and proteomic levels in the present study, as shown in Supplementary Figure 3. Some of the key enzymes involved in the biosynthesis and polymerization of lignin (such as caffeoyl CoA *O*-methyltransferase, 4-coumarate-CoA ligase, cynnamoyl-CoA dehydrogenase, and laccase) accumulated in the leaves and roots of infected plants. The upregulation of four dirigent-like proteins, which are part of the machinery that builds extracellular lignin-based structures^74^, was also observed in both the leaves and roots. Genes and proteins involved in isoprenoid biosynthesis were downregulated in both the leaves and roots, while the biosynthesis of flavonoids appeared to be upregulated. Lastly, enzymes involved in alkaloid biosynthesis, such as strictosidine synthase and tropinone reductase, were upregulated in the roots.

We observed the alteration of several enzymes involved in cell wall remodeling after *X. translucens* invasion in leaves, including polygalacturonases, alpha- and beta-glucosidases, alpha-fucosidase, beta-D-xylosidase, and pectin acetylesterase. Cell wall remodeling may affect intracellular signal transduction and the cell defense response at the site of pathogen attack. In infected leaves, we also observed accumulation of an auxin-inducible expansin. Expansins, which mediate long-term extension of the cell wall, can render the plant cell wall vulnerable, creating an opportunity for pathogen attack. Xylan is the major hemicellulose polymer in cereals. Infected leaves can counteract microbial endoxylanases by accumulating xylanase inhibitors, as shown by our RNAseq and proteomic data. In the roots, the accumulation of proteins involved in cell wall degradation (one endo-beta-mannosidase, one β-1,4-glucanase, and one polygalacturonase) suggests a change in root development upon foliar attack.

In conclusion, foliar attack by *X. translucens* increases secondary metabolite concentrations in roots to a similar extent as observed locally in leaves. Similar results were observed in plants attacked by leaf herbivores^3, 4^. It was proposed that synthesis of defense proteins in roots upon foliar infection may allow plants to continue to protect themselves, even when large parts of the aboveground components of the plant are destroyed. Moreover, the fact that roots allow access to nitrogen and phosphate in the soil may mean that the production of certain secondary metabolites in roots is advantageous^4^. The upregulation of nitrogen transporters and nitrate reductase in the roots in our experiments supports this hypothesis. Several studies showed that secondary metabolites and cysteine proteases that were upregulated in roots were synthesized in the roots and transported to the shoot to provide resistance to foliar attack^75^.

## Conclusion

**Figure 6:**
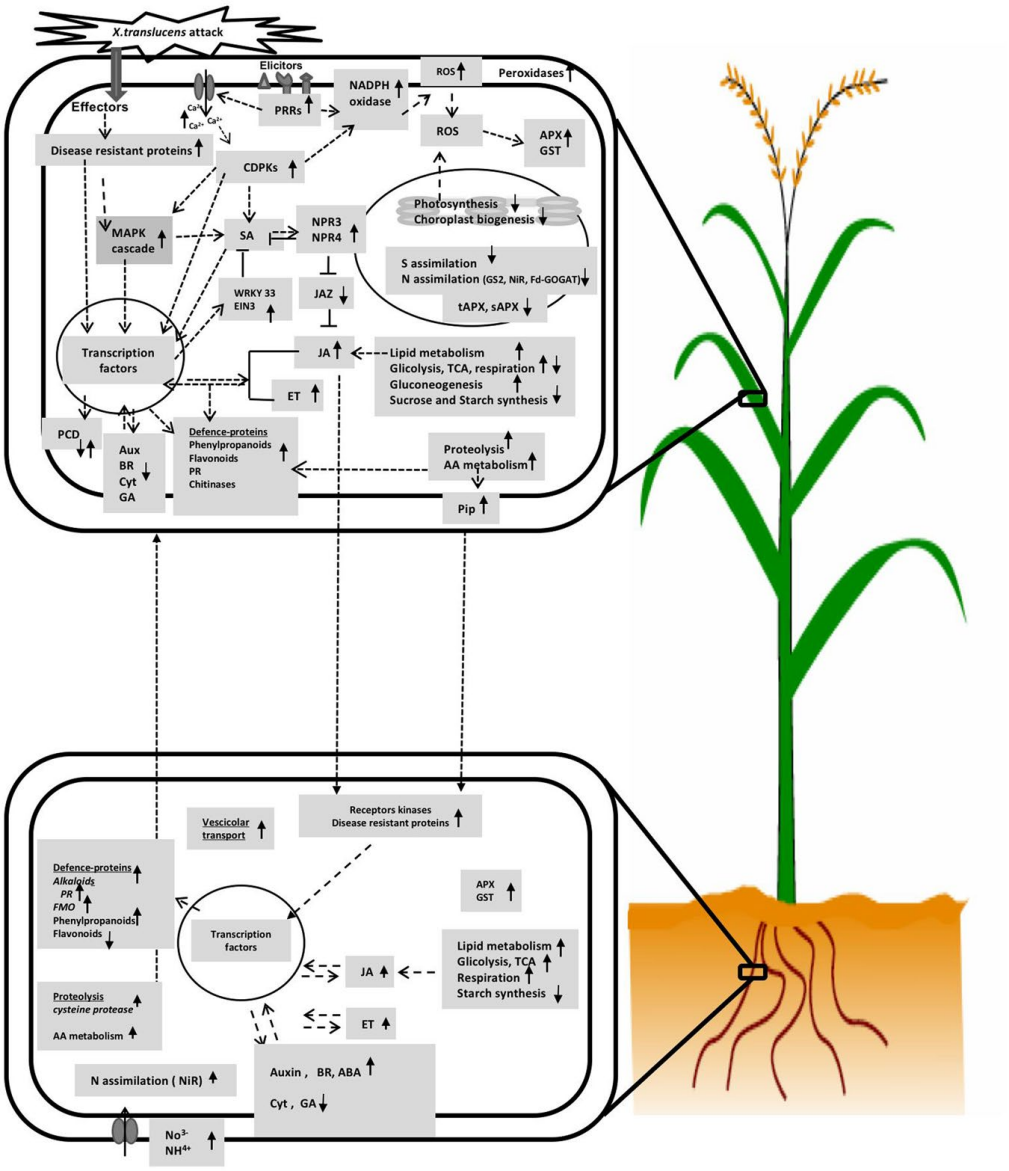
Proposed systemic response mechanism of wheat towards infection by *X. translucens*.

The objective of this study was to obtain molecular information about the local and systemic responses triggered by *X. translucens* infection in wheat plants (Fig. 6). The following important points emerged:

1. *X. translucens* activates a large recognition platform around the infection site that contributes to a more rapid achievement of the signaling threshold needed for PTI activation.
2. Chloroplasts are strategic battlefields during *X. translucens* attack.
3. *X. translucens* attack induces an active interplay between SA and JA.
4. Diverse biological processes are integrated to optimize plant fitness during pathogen infection.
5. Exposure to *X. translucens* for 24 h induces a substantial response both in local (infected leaves) and distant (roots) tissues. Accumulation of JA, PIP, and/or the downstream mediator of H_2_O_2_ could act as a long-distance signal from infected leaves to roots.
6. Systemic root responses significantly differ from those triggered in infected leaves, and, in several cases, these responses are in complete contrast to the responses in leaves. Roots do not only detect foliar attack but seemingly also play an important role in the defense by acting as a site of energy production and synthesis for various secondary metabolites. Moreover, the activation of defenses in roots following foliar attack might change the root response to subsequent pathogens and/or the colonization level and composition of root-associated microbes.

## Electronic supplementary material

Supplementary data legends

Table S1

Table S2

Table S3

Table S4

Table S5

Table S6

Table S7

Table S8

Table S9

Supplementary Dataset 10.
