# Supplementary material for: Transcriptome and proteome analysis reveal new insight into proximal and distal responses of wheat to foliar infection by Xanthomonas translucens
Source: Sci Rep. 2017 Aug 31;7:10157. doi: 10.1038/s41598-017-10568-8 (PMC5579275; doi:10.1038/s41598-017-10568-8)
Supplement: Supplementary file 1 — Supplementary data legends [file 41598_2017_10568_MOESM1_ESM.doc]

**Transcriptome and proteome analysis reveal new insight into proximal and distal responses of wheat to foliar infection by *Xanthomonas translucens***

Garcia-Seco, D., Chiapello, M., Bracale, M., Pesce, C., Bagnaresi, P., Dubois, E., Moulin, L., Vannini, C., Koebnik, R.

**SUPPLEMENTAL DATA LEGENDS**

**TABLES**

**Supplementary Tables**

**Table S1**

Details of raw reads and mapped reads. Samples are referred to leaves and roots of control and *X. translucens*-infected plants.

UNIQUE : Number of reads that align just in one position (criteria : MAPQ >= 3)

CAF : Number of filtered clusters

MULTI : Number of reads that align just in several positions (criteria : MAPQ >= 3)

UNMAPPED : Number of reads that no align

**Table S2**

Expression values of all detected transcribed genes in leaves (sheet 1) and roots (sheet 2) reported as DESeq-normalized read counts and log2 fold changes.

Id (transcript id), baseMean (expression level or mean of normalized counts), log2FoldChange (logarithmic [base 2] fold-change in read abundance/expression level in treatment over control (> 0 is enrichment in treatment, < 0 is decrease in treatment), lfcSE (logfoldStandard Error), stat (Wald statistic), pvalue (Wald test p-value) padj (adjusted p-values), Gene.stable.ID, Gene.name, Gene.description, Chromosome.scaffold.name, Transcript.start..bp, Transcript.end..bp., GO_tutte_info_IWGSC_1.0_popseq_2.2 (GO term in IWGSC).

**Table S3**

Up-regulated and down-regulated genes in leaves (sheet 1) and roots (sheet 2) of wheat plants infected with *X. translucens* for 24h. Tables are sorted according to fold change.

Id (transcript id), baseMean (expression level or mean of normalized counts), log2FoldChange (logarithmic [base 2] fold-change in read abundance/expression level in treatment over control (> 0 is enrichment in treatment, < 0 is decrease in treatment), lfcSE (logfoldStandard Error), stat (Wald statistic), pvalue (Wald test p-value) padj (adjusted p-values), Gene.stable.ID, Gene.name, Gene.description, Chromosome.scaffold.name, Transcript.start..bp, Transcript.end..bp., GO_tutte_info_IWGSC_1.0_popseq_2.2 (GO term in IWGSC).

**Table S4**

Comparison of DEGs between leaves and roots.

Id (transcript id), log2FoldChange_leaves (logarithmic [base 2] fold-change between control and treated samples in leaves), padj_leaves (adjusted p-values in leaves), log2FoldChange_roots (logarithmic [base 2] fold-change between control and treated samples in roots), padj_roots (adjusted p-values in roots), reg_leaves (indicates if the transcript is regulated: 1 means that the transcript is up-regulated; 0 means that the transcript is not-regulated;-1 means that the transcript is down-regulated), reg_roots (as for leaves), ind (indicate the type of regulation: 0 not regulation; 1 up-regulated only in roots; 2 down-regulated only in roots; 3 up-regulated only in leaves; 4 down-regulated only in roots; 5 up-regulatet both in roots and leaves; 6 down-regulated both in roots and leaves; 7 up-regulated in roots and down-regulated in leves; 8 down-regulated in roots and up-regulates in leaves.

**Table S5**

Transcriptomics MapMan identifications.

BinCode (MapMan id code), BinName (MapMan id name), id (transcript identifier), description (transcript name), log2FC (logarithmic [base 2] fold-change)

**Table S6**

Identified proteins in wheat roots and leaves.

Protein.IDs2 (Leading group protein ID), At_Protein.IDs (Leading group protein best match on *A. thaliana* database), At_Desc (Leading group protein description based on *A. thaliana* database), xan* (3 column containing the treated values log2 transformed), control* (3 column containing the control values log2 transformed), input_t (Indicates the imputed proteins in treated sample: 0 means that the software did not impute any values; 1 means thtat the software imputed all values missing at random (technical meaning); 2 means that the software imputed all values missing NOT at random (biological meaning) – proteins absent in treated samples), imput_c (indicates the imputed proteins in control sample: 0 means that the software did not impute any values; 1 means that the software imputed all values missing at random (technical meaning); 2 means that the software imputed all values missing NOT at random (biological meaning) – proteins absent in control samples), xantho (treated sample mean value), control (control sample mean value), pval (pvalue as calculated by the t-test), qval (corrected pvalue [FDR]), logFC (fold-change expressed in logarithmic scale [base 2]), reg (indicates if the protein is regulated: 1 means that the protein is up-regulated ; 0 means that the protein is not-regulated ;-1 means that the protein is down-regulated), Protein.IDs (all group protein IDs).

**Table S7**

Compare DEPs between leaves and roots.

Protein.IDs2 (protein id), logFC_leaves (logarithmic fold-change between control and treated samples in leaves), qval_leaves (adjusted p-values in leaves), logFC_root (logarithmic fold-change between control and treated samples in roots), qval_root (adjusted p-values in roots), reg_leaves (indicates if the protein is regulated: 1 means that the protein is up-regulated ; 0 means that the protein is not-regulated ;-1 means that the protein is down-regulated), reg_roots (as for leaves), ind (indicate the type of regulation: 0 not regulation; 1 up-regulated only in roots; 2 down-regulated only in roots; 3 up-regulated only in leaves; 4 down-regulated only in roots; 5 up-regulatet both in roots and leaves; 6 down-regulated both in roots and leaves; 7 up-regulated in roots and down-regulated in leves; 8 down-regulated in roots and up-regulates in leaves.

**Table S8**

Proteomics MapMan identifications.

BinCode (MapMan id code), BinName (MapMan id name), id (MapMan protein identifier), description (protein name), log2FC (Fold change)

**Table S9**

It contains all the common proteins/genes identified in proteomics and transcriptomics experiment in roots.

Prot_id (protein id), qval (adjusted p-values), logFC (logarithmic fold-change between control and treated samples), reg (indicates if the protein is regulated: 1 means that the protein is up-regulated; 0 means that the protein is not-regulated; -1 means that the protein is down-regulated), id (transcript id), log2FoldChange (logarithmic [base 2] fold-change between control and treated samples), padj (adjusted p-value), identity (the extent to which two (nucleotide or amino acid) sequences have the same residues at the same positions in an [alignment](http://www.ncbi.nlm.nih.gov/books/NBK62051/def-item/alignment/), often expressed as a percentage), evalue (represents the number of different alignments with scores equivalent to or better than S that is expected to occur in a database search by chance), bit_score (the bit score, S', is derived from the raw alignment score, S, taking the statistical properties of the scoring system into account), treg (indicates if the transcript is regulated: 1 means that the transcript is up-regulated; 0 means that the transcript is not-regulated; -1 means that the transcript is down-regulated), Desc (leading group protein description).

**Supplementary Dataset 10.**

Total protein carbonylation level in Triticum plants.

Carbonylated proteins were detected by anti-2,4 – dinitrophenol (DNP) immunoassay in Leaves (A) and roots (B) of control plants (C) or mycorrized (M) plants and in the presence of Xanthomonas infection (X, control plants + Xanto, M-X, mycorrized plant + Xanto). Coomasie blue staining is shown as loading control.
