# Supplementary material for: Transcriptome and proteome analysis reveal new insight into proximal and distal responses of wheat to foliar infection by Xanthomonas translucens
Source: Sci Rep. 2017 Aug 31;7:10157. doi: 10.1038/s41598-017-10568-8 (PMC5579275; doi:10.1038/s41598-017-10568-8)

**Transcriptome and proteome analysis reveal new insight into proximal and distal responses of wheat to foliar infection by *Xanthomonas translucens***

Garcia-Seco, D., Chiapello, M., Bracale, M., Pesce, C., Bagnaresi, P., Dubois, E., Moulin, L., Vannini, C., Koebnik, R.


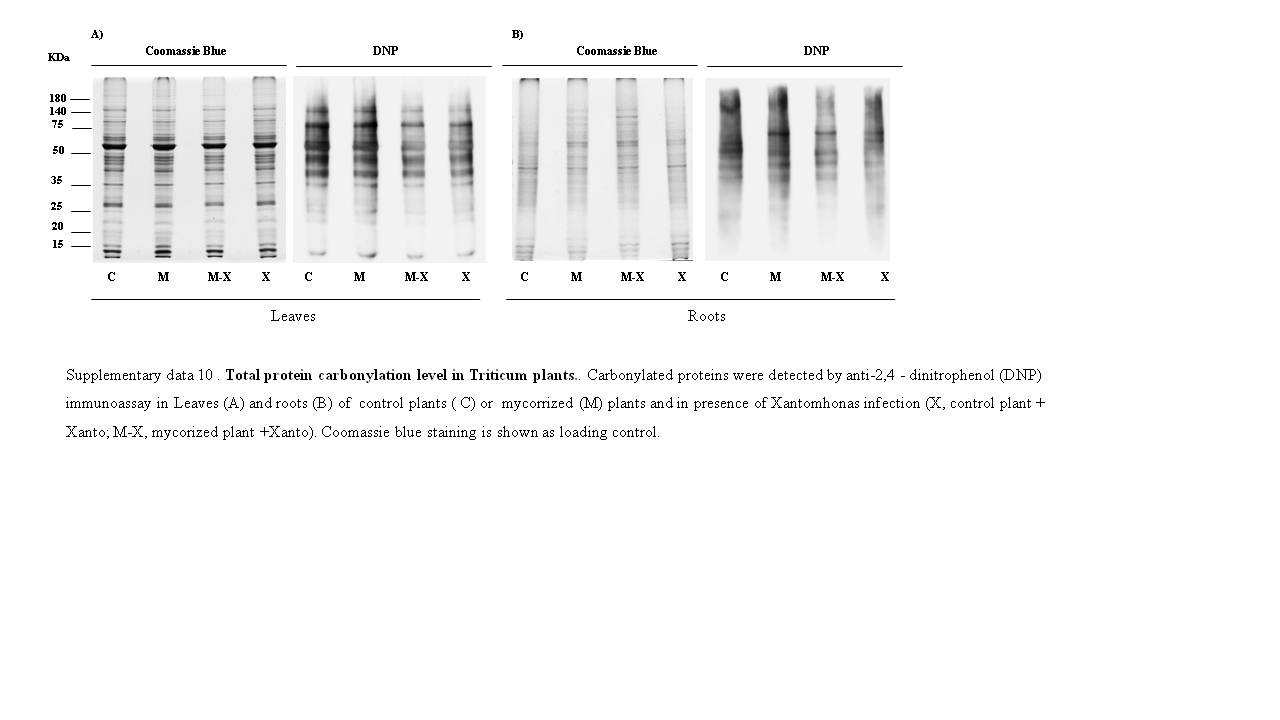

Supplement: Supplementary file 11 — Supplementary Dataset 10. [file 41598_2017_10568_MOESM11_ESM.doc]
